# Unraveling the Role of Inwardly Rectifying Potassium Channels in the Hippocampus of an Aβ_(1–42)_-Infused Rat Model of Alzheimer’s Disease

**DOI:** 10.3390/biomedicines8030058

**Published:** 2020-03-13

**Authors:** Enes Akyuz, Chiara Villa, Merve Beker, Birsen Elibol

**Affiliations:** 1 Department of Biophysics, Faculty of Medicine, Yozgat Bozok University, Yozgat 66100, Turkey; 2 School of Medicine and Surgery, University of Milano-Bicocca, 20900 Monza, Italy; 3 Department of Medical Biology, Faculty of Medicine, Bezmialem Vakif University, Istanbul 34093, Turkey; mbeker@bezmialem.edu.tr (M.B.); bcan@bezmialem.edu.tr (B.E.)

**Keywords:** Alzheimer’s disease, amyloid beta, hippocampus, Kir channels, K^+^ channels

## Abstract

Alzheimer’s disease (AD) is a progressive neurodegenerative disorder with a complex etiology and characterized by cognitive deficits and memory loss. The pathogenesis of AD is not yet completely elucidated, and no curative treatment is currently available. Inwardly rectifying potassium (Kir) channels are important for playing a key role in maintaining the resting membrane potential and controlling cell excitability, being largely expressed in both excitable and non-excitable tissues, including neurons. Accordingly, the aim of the study is to investigate the role of neuronal Kir channels in AD pathophysiology. The mRNA and protein levels of neuronal Kir2.1, Kir3.1, and Kir6.2 were evaluated by real-time PCR and Western blot analysis from the hippocampus of an amyloid-β(Aβ)_(1-42)_-infused rat model of AD. Extracellular deposition of Aβ was confirmed by both histological Congo red staining and immunofluorescence analysis. Significant decreased mRNA and protein levels of Kir2.1 and Kir6.2 channels were observed in the rat model of AD, whereas no differences were found in Kir3.1 channel levels as compared with controls. Our results provide in vivo evidence that Aβ can modulate the expression of these channels, which may represent novel potential therapeutic targets in the treatment of AD.

## 1. Introduction

Alzheimer’s disease (AD; MIM#104300) is a chronic irreversible neurodegenerative disorder and represents the most common form of dementia in elderly individuals [1]. AD is clinically characterized by a progressive memory deterioration, thinking difficulty, confusion, and changes in personality, behavior, and language, resulting in autonomy loss that finally requires full-time medical care [2]. The neuropathological hallmarks of AD include the presence of extracellular senile plaques constituted by the amyloid-β (Aβ) peptide and intracellular neurofibrillary tangles (NFTs) composed of hyper-phosphorylated paired helical filaments of the microtubule-associated protein tau (MAPT) [3]. Among Aβ species, Aβ_(1–42)_, which is generated from Aβ precursor protein (APP) sequentially cleaved by β-secretase and γ-secretase, is considered more toxic than Aβ_(1–40)_ because of its strong tendency to aggregate [4]. The Aβ deposition in the brain triggers a series of neurodegenerative processes, including synaptic toxicity, microglia-mediated inflammation, mitochondrial dysfunction, and oxidative stress, which in turn lead to cell death [5]. Furthermore, Aβ pathogenesis reduces the synthesis of acetylcholine (ACh) and negatively affects the acetylcholinesterase (AChE) activity [6]. Despite its prevalence, the AD pathogenesis is not completely understood, and, currently, there are no effective treatments to slow or halt the progression of its symptoms [7].

Emerging evidence points out a key role of ion channels in the progress and development of a variety of neurological disorders, including epilepsy [8,9], autism spectrum disorders [10], multiple sclerosis [11], and AD [12]. Among them, the inwardly rectifying potassium (K^+^) channels (Kir) are essential for maintaining the resting membrane potential and controlling the cell excitability by the regulation intracellular and extracellular flow of K^+^ ions in different types of cells, including neurons. To date, seven subfamilies (Kir1-Kir7) have been identified according to their sequence similarity and function properties [13]. An important involvement of Kir2.x, Kir3.x, and Kir6.x channels in the pathogenesis of AD has been supported by both in vitro and in vivo models [11]. Evidence showed an impaired activity or an altered expression of these channels, probably modulated by Aβ [12,14]. Given the limitations of investigating AD in human subjects, current studies mostly rely on animal models in order to understand the underlying molecular mechanisms of this disorder. Some experimental in vivo models mimicking the major neuropathological hallmarks in AD have already been developed for studying Kir channels in the disease pathogenesis [12,15], but no data are available regarding an Aβ_(1–42)_-infused rat model of AD.

Herein, we aim to better elucidate the role of Kir2.1, Kir3.1, and Kir6.2 channels in AD pathophysiology by analyzing their mRNA and protein levels in the hippocampus of an Aβ_(1–42)_-infused rat model of AD. This represents a valuable tool that recapitulates some key features of human AD, including Aβ plaque, cholinergic dysfunction, neuron loss, ventricular enlargement, and behavior deficiencies.

## 2. Materials and Methods

### 2.1. Animals

Adult female Sprague–Dawley rats (6-month-old; *n* = 14) were housed in a quiet, temperature and humidity-controlled room (21 ± 2 °C; 62% ± 7% relative humidity; 12-h cycles dark/light). Rats were fed ad libitum with a standard dry rat diet and tap water. All procedures were carried out in strict accordance with the recommendations in the Guide for the Care and Use of Laboratory Animals adopted by the National Institutes of Health (NIH, Bethesda, MD, USA) and the Declaration of Helsinki. Experimental protocol of this study was approved by the local scientific ethical committee of Bezmialem Vakif University, Istanbul, Turkey (2015/229). All efforts were made to minimize animal suffering.

### 2.2. Aβ_(1–42)_-Infused Rat Model

A solvent of 35% acetonitrile plus 0.1% trifluoroacetic acid was used to reconstitute the Aβ_(1–42)_ peptide (SCP0038, Sigma-Aldrich, St. Louis, MO, USA) and soluble peptide suspensions were incubated at 37 °C for 72 h with gentle shaking for fibril formation. The rats were injected intra-cerebroventricularly (ICV) with oligomeric Aβ_(1–42)_ to induce AD. Briefly, after seven days of acclimation, rats were anesthetized with an intraperitoneal injection of a ketamine and xylazine mixture (100 and 10 mg/kg body weight, respectively) and then placed in a stereotaxic apparatus. A stainless steel cannula was stereotaxically implanted into the right hippocampus of rats (coordinates from bregma: −3.60 mm anteroposterior; −2.00 mm lateral; −4.00 mm vertical) and fixed to the skull with dental cement. A mini-osmotic pump (Alzet 2002, Durect, Cupertino, CA, USA) was attached and implanted subcutaneously near the scapula for a continual infusion.

### 2.3. Experimental Design

Rats that underwent ICV infusion were randomly divided into two groups (*n* = 7 per group): (i) sham control that received injections of 0.9% NaCl saline solution, and (ii) Aβ_(1–42)_-infused group injected with Aβ_(1–42)_ oligomers at the rate of 300 pmol/day for 14 days. Rats were sacrificed with decapitation after a 14-day infusion. The brains were quickly removed, and their both right and left hippocampi were dissected and then stored at −80 °C until molecular analysis.

### 2.4. Histological Congo Red Staining

Coronal sections from the hippocampus were prepared at 20 μm thickness using a cryostat and fixed in ice-chilled 4% paraformaldehyde (PFA). For labeling Aβ deposits, slices were stained with 1% Congo red solution (Sigma-Aldrich, St. Louis, MO, USA) in 80% of absolute ethanol and 1% of NaOH. After being washed, sections were counterstained with cresyl violet, dehydrated in absolute ethanol, and then cleared in xylene. Specimens were mounted on slides and evaluated under a light microscope (Nikon Microscopy, Tokyo, Japan). For quantification, images were analyzed by color segmentation plugin–ImageJ software (NIH, Bethesda, MD, USA). The entire area of deposits was considered.

### 2.5. Immunofluorescence Analysis

The PFA-fixed slices were blocked with 10% normal goat serum for 1 h. Sections were immunostained with the application of 1:100 dilution of primary anti-Aβ rabbit polyclonal antibody (8243, Cell Signaling Technology, Danvers, MA, USA) followed by goat anti-rabbit Alexa Fluor^®^ 488 conjugated secondary antibody (A11034, Thermo Fisher Scientific, Waltham, MA, USA) at 1:200 dilution. Nuclei were marked blue with 40,6-diamidino-2-phenylindole (DAPI). The sections were mounted on slides and evaluated under a fluorescence microscope (Axio, Zeiss, Germany).

### 2.6. cDNA Synthesis and Real-Time PCR

Total RNA was isolated from homogenized hippocampal tissue with TRIzol and PureLink RNA mini kit (Thermo Fisher Scientific, Waltham, MA, USA), according to the manufacturer’s instructions. One microgram of the total extract amount of RNA was treated with DNase I and reverse-transcribed using High Capacity cDNA Reverse Transcription Kit according to the manufacturer’s suggested protocol (Applied Biosystems, Foster City, CA, USA). The first-strand cDNA was used as a template for real-time PCR (RT–PCR) using rat specific primers for *Kcnj2* (Kir2.1), *Kcnj3* (Kir3.1), and *Kcnj11* (Kir6.2), as reported in Table 1. RT–PCR reaction was performed with the SYBR Green PCR kit (iTaq™ Universal SYBR^®^ Green, Biorad, Hercules, CA, USA) using a CFX96 real-time system sequence detector (Biorad, Hercules, CA, USA). Data, normalized to the housekeeping control gene (*Gapdh*), are expressed as fold change values respect to the sham control group according to the 2^-ΔΔCt^ algorithm, as previously described [16].

### 2.7. Western blotting (WB)

Total protein extracts were obtained by lysing 0.25 g hippocampal tissue with 1 X RIPA lysis buffer (50 mM Tris-HCl pH 7.4, 150 mM NaCl, 1% Triton X-100, 0.1 % SDS) added with 1 mM DTT, 1 mM EDTA and EGTA, and 1.5% protease inhibitor cocktail and phosphatase inhibitor cocktail. Total protein concentration was measured using the Pierce BCA Protein Assay Kit (Thermo Fisher Scientific, Waltham, MA, USA) by a Multiskan™ GO Microplate spectrophotometer. Equal amounts of proteins were boiled for 5 min and separated by SDS–PAGE followed by transfer to PVDF membrane. Then, the membranes were blocked with 5% milk solution prepared in TBST (Tris-buffered saline, 0.1% Tween 20) buffer and incubated with one of the following primary antibodies against Kir2.1 (rabbit polyclonal, 1:200, Abcam, Cambridge, UK [ab65796]), Kir3.1 (mouse monoclonal, 1:200, Alomone Labs, Jerusalem, Israel [APC-005]), Kir6.2 (rabbit polyclonal, 1:200, Alomone Labs, Jerusalem, Israel [APC-020]), or β-actin (mouse monoclonal, 1:5000, Thermo Fisher Scientific, Waltham, MA, USA [AC-15]). Membranes were washed three times in TBST and then incubated with ECL anti-mouse or anti-rabbit horseradish peroxidase-conjugated IgG secondary antibodies (1:5000, GE Healthcare Life Sciences, Amersham, UK). The protein bands were developed with luminol-based substrate (Advansta, San Jose, CA, USA) and chemiluminescent signal was digitally acquired by CCD camera with Fusion FX7 (Vilber Lourmat, France) system. Densitometric analysis of Western blot bands was performed using the “gel analyzer” function of ImageJ software (NIH, Bethesda, MD, USA).

### 2.8. Statistical Analysis

Data are generally given as mean values ± standard error of the mean (SEM). Pairwise comparisons were performed by Mann–Whitney U-test. The version 18 of Statistical Package for Social Science (SPSS 18, IBM Corporation, Chicago, IL, USA) was used for statistical analysis of the data. Differences were considered significant at * *p* < 0.05 and ** *p* < 0.01.

## 3. Results

### 3.1. Injection of Aβ_(1–42)_ Oligomers Mimicked Alzheimer’s Disease in Rats

**Figure 1:**
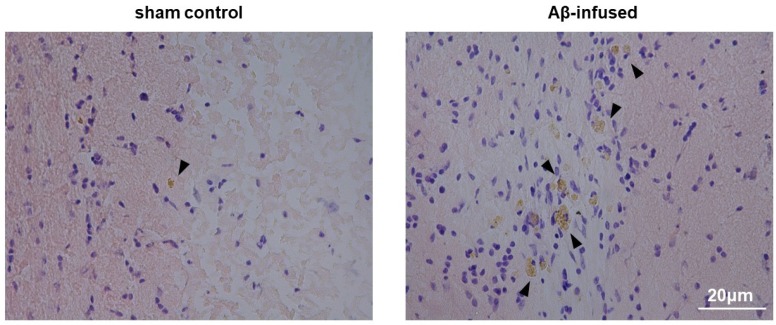
Representative images of hippocampus sections stained with Congo red in sham control and Aβ_(1–42)_-infused rat model. Extracellular Aβ deposits were visualized in brownish color and indicated by arrows; the nuclei were counterstained with cresyl violet (purple). Pictures were taken at the magnification of ×20. Scale bar: 20 µm.

**Figure 2:**
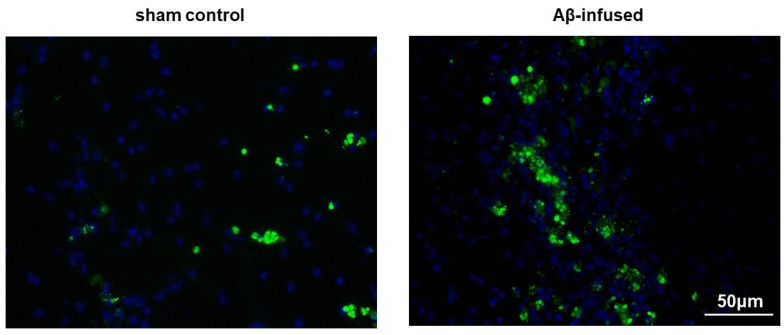
Representative images of hippocampus section stained with anti-Aβ antibody (green fluorescence) in sham control and Aβ_(1–42)_-infused rat model. Cell nuclei were counterstained with DAPI (blue fluorescence). Scale bar: 50 µm.

As a result of Aβ_(1–42)_ infusion for 14 days, both Congo red histological staining and immunofluorescence analysis confirmed the extracellular presence of oligomeric and aggregated forms of Aβ in the hippocampus of rat model as compared with the sham control (2.73-fold change over sham controls, * *p* < 0.05, Figure 1; 2.21-fold change over sham control, * *p* < 0.05, Figure 2, respectively).

### 3.2. Aβ_(1–42)_-Infused Rats Exhibited Low mRNA Levels of Neuronal Kir2.1 and Kir6.2 Channels

With the purpose of investigating a possible role of neuronal Kir channels in AD pathogenesis, we firstly analyzed mRNA levels of Kir2.1, Kir3.1, and Kir6.2 channels in both ipsilateral and contralateral hippocampi from rats by RT–PCR.

**Figure 3:**
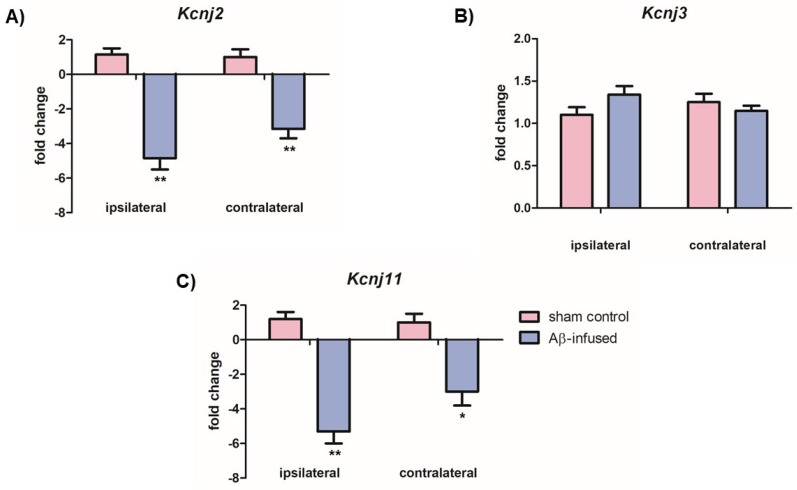
Relative mRNA levels of (**A**) *Kcnj2* (Kir2.1), (**B**) *Kcnj3* (Kir3.1), and (**C**) *Kcnj11* (Kir6.2) in ipsilateral and contralateral hippocampi from both sham control and Aβ_(1–42)_-infused rat model. Data are expressed as fold change of mRNA levels normalized to the housekeeping control gene (*Gapdh*) and represent the mean ± SEM obtained in 3 independent experiments, *n* = 7 for each group, * *p* < 0.05, ** *p* < 0.01.

Significantly decreased mRNA levels of Kir2.1 (*Kcnj2*) and Kir6.2 (*Kcnj11*) channels were observed in both ipsilateral and contralateral hemispheres of Aβ_(1–42)_-infused rats as compared with sham controls (Figure 3A: 4.85-fold change and 3.15-fold change over controls, ** *p* < 0.01; Figure 3C: 5.30-fold change and 3.00-fold change over controls, ** *p* < 0.01 and * *p* < 0.05, respectively). However, no significant differences were found in mRNA levels of the Kir3.1 (*Kcnj3*) channel in both hemispheres (Figure 3B, *p* > 0.05).

### 3.3. Low mRNA Levels of Kir2.1 and Kir6.2 Channels Correlate with Decreased Protein Levels in Aβ_(1–42)_-Infused Rat Model

In order to assess if changes in mRNA levels of Kir channels result in different protein levels, total protein extract from both ipsilateral and contralateral hippocampus tissues were analyzed via WB analysis by using specific anti-Kir antibodies and the relative protein abundance was quantified by densitometric measurements.

**Figure 4:**
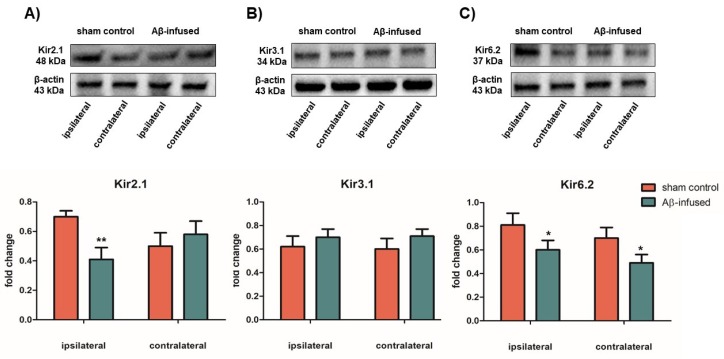
Protein expression levels of Kir2.1 (**A**), Kir3.1 (**B**), and Kir6.2 (**C**) channels in ipsilateral and contralateral hippocampi from both sham control and Aβ_(1–42)_-infused rat model. Upper panel: representative images of WB analysis on total protein extracts. β-actin was used as endogenous control for equal protein load. Lower panel: densitometric analysis of Kir2.1 (**A**), Kir3.1 (**B**), and Kir6.2 (**C**) protein levels. Data are expressed as fold change ratio on sham control and normalized to the β-actin protein levels. Bars represent the mean ± SEM obtained in 3 independent experiments, *n* = 7 for each group, * *p* < 0.05, ** *p* < 0.01.

Significantly decreased Kir2.1 protein levels were detected only in the ipsilateral hemisphere of Aβ_(1–42)_-infused rats as compared with sham controls (Figure 4A: 0.41 ± 0.50 vs. 0.72 ± 0.25, ** *p* < 0.01). Decreased Kir6.2 protein levels were observed in both ipsilateral and contralateral hippocampus tissues of Aβ_(1–42)_-infused rats as compared with sham controls (Figure 4C: 0.61 ± 0.35 vs. 0.82 ± 0.52, * *p* < 0.05, and 0.52 ± 0.25 vs. 0.71 ± 0.40, * *p* < 0.05, respectively). On the other hand, no significant differences in Kir3.1 protein levels were found in Kir3.1 protein levels in both hemispheres (Figure 4B, *p* > 0.05), confirming previous mRNA data (Figure 3B).

## 4. Discussion

Functional and expression alterations of K^+^ channels cause disruptions in neuronal balance and membrane excitability, contributing to the development and progress of several neurological diseases, including AD [8,9,10,11,12]. Among them, Kir channels have the ability to mediate the inward flow of K^+^ ions at hyperpolarizing membrane voltages more readily than the outward flow of K^+^ at depolarizing voltages [13]. They are involved in a number of essential physiological processes, such as the regulation of hormone secretion, generation of electrical impulses, and control of vascular smooth muscle tone. It is known that a variety of severe human disorders are directly related to a dysfunction of Kir channel proteins [17]. Moreover, intracellular Na^+^ and K^+^ levels were found to be increased in brain regions of AD patients, pointing out a cellular ion imbalance in AD pathophysiology [18]. So, given their function in maintaining the resting membrane potential and K^+^ homeostasis of most cells [13], we aim to highlight the role of neural Kir channels in AD by analyzing their mRNA and protein levels in the hippocampus of Aβ_(1–42)_-infused rat model of the disease.

The classical Kir2 subfamily exhibits a strong inward rectifying property and it is the major responsible for the *I*_K1_ current. Kir2.1 channels hyperpolarize the cells in response to an increase in the external K^+^ concentration [19]. Our data showed a decrease in both mRNA and protein levels of Kir2.1, suggesting a reduced Kir current in the hippocampus of AD model rats. We can speculate that Aβ peptide may decrease the expression of this channel, affecting the hippocampal activity balance underlying memory and learning processes damaged in AD [14]. However, other authors reported no differences in Kir2.1 mRNA expression in the hippocampus of rats with cholinergic impairment, probably because of the use of different models [15].

In addition to Kir2.1, a decrease in both transcript and protein levels of Kir6.2 channel has also been observed. In contrast with *I*_K1_ channels, Kir6 (also known as adenosine triphosphate (ATP)-sensitive K^+^, K_ATP_) subfamily are weakly inwardly rectifying and are inhibited by intracellular ATP levels [13]. They correlate the metabolic status of neurons to their excitability by detecting changes of intracellular phosphate potential (e.g., ATP/ADP ratio) [20]. Functional channels consist of four pore-forming Kir6 subunits (Kir6.1 and Kir6.2) and four sulfonylurea receptor (SUR) subunits (SUR1, SUR2 A, and SUR2 B). In neurons, the K_ATP_ channels are mainly constituted by the coassembly of Kir6.2/SUR1 subunits [13]. They are also involved in the generation of the glucose-sensitive K^+^ current in neurons, indicating that the increase in neuronal excitation observed when the concentration of external glucose raises is due to the closure of K_ATP_ channels [21]. It is well-known that the Kir6 subfamily is involved in AD pathogenesis and phenotype [12]. The first evidence has been addressed by a study in which increased transcript levels of Kir6.1 were observed in the hippocampus of cholinergic impaired rats, whereas mRNA expression of Kir6.2 was significantly increased in the cortex [15]. Consistent with these findings, high Kir6.2 protein levels were also found in both hippocampal reactive astrocytes from a triple transgenic mouse model of AD (3 xTg-AD) [22]. On the other hand, we found a decrease in both mRNA and protein levels of Kir6.2 in the hippocampus of Aβ_(1–42)_-infused rats. These contrasting results may be due to the use of different AD models. Interestingly, the transgenic overexpression of the Kir6.2 channel in the forebrain protects mice from neuronal damage and hypoxic–ischemic injury seen in stroke [23]. Moreover, it has been shown that Kir6.2 knock-out mice showed severe deficits in long-term memory processes and learning [24,25]. Therefore, we can hypothesize that the impairment of memory occurring in AD may be related to a downregulation of the Kir6.2 subunit.

Concerning Kir3.1 channels, no statistical differences were found. Also named as G-protein-coupled Kir (GIRK) channels, they are activated by some neurotransmitters (e.g., acetylcholine GABA, dopamine) through the stimulation of their G protein coupled receptors (GPCRs), resulting in a reduced action potential firing and a cell membrane hyperpolarization. GIRK channels are detected in the extra-synaptic membrane of CA1 hippocampal pyramidal neurons and play a role in the production of slow inhibitory post-synaptic potential [13]. However, our data did not show any significant differences in either transcript or protein levels of Kir3.1 channel in both hemispheres of hippocampus from the Aβ_(1–42)_-infused rat model, in line with an already reported study [15]. We can speculate that this evidence may be due to two contrasting effects of Aβ on these channels in a more complex neuronal network. Indeed, it has been reported that this peptide led to a GIRK3 channel upregulation, which resulted in K^+^ efflux from neurons triggering, thus, the Aβ-mediated apoptotic pathway [26]. On the contrary, other authors reported an opposite effect of Aβ in which it modulated GIRK3 expression by downregulating these channels [14].

In summary, our data support the evidence that Aβ can modulate the expression of neuronal Kir channels in the AD pathogenesis. The fact that we reported some results that are contrasting with the previous ones may be related to the use of different in vivo models that recapitulate distinct features of the disorder. Indeed, due to the lack of complete understanding of AD etiology, the development of adequate animal models resembling all stages of disease progression, as well as the merging convergent pathways of neurodegeneration, still represents a need for AD research. However, the complementary use of several models will help to understand molecular mechanisms underlying the disease and to develop novel strategies based on the modulation of Kir channels or their accessory subunits for AD prevention and therapy.

## 5. Conclusions

Overall, our data corroborate the working hypothesis that Kir channels play a causative role in AD pathogenesis, as suggested by their altered mRNA and protein levels found in the Aβ_(1–42)_-infused rat model. However, it cannot be excluded a complex mechanism of Aβ, which makes such reported alterations the result of an impaired metabolic pathway involving related channels or other proteins. We are aware that our study has several limitations, including the confirmation that differences observed in protein amounts are translated into an altered channel function activity by patch-clamp recordings and the lack of tests assessing learning and memory deficits. Moreover, it should be noted that, although cerebral infusion of Aβ in rats can recapitulate some hallmarks of human AD, it cannot properly reproduce the progressive neurodegeneration occurring during the disease, so further studies in different models are necessary to cover all aspects of the disease.

## Figures and Tables

**Table 1:** List of primers for RT–PCR.

Genes	Forward Primer (5′–3′)	Reverse Primer (5′–3′)
*Kcnj2*	GCAAACTCTGCTTGATGTGG	TCATACAAAGGGCTGTCTTCG
*Kcnj3*	CTGACCGCTTCACATAGC	CTCCAGACTGGGATAGAC
*Kcnj11*	CCTACACCAGGTGGACATCC	CAGGCTGCGGTCCTCATCAA
